# Defect-Driven Optical Modulation in Rare-Earth-Modified Zn_2_SnO_4_ Spinels for Advanced Optoelectronic Applications

**DOI:** 10.1021/acsomega.5c13376

**Published:** 2026-05-26

**Authors:** Ramesh Kumar Raji, Noor S. Alnahdi, Tasnem Hamam, Naser Qamhieh, Adel Najar, Falah Awwad, Saleh T. Mahmoud

**Affiliations:** † Department of Physics, College of Science, 11239 United Arab Emirates University, Al-Ain, P.O. Box Abu Dhabi 15551, United Arab Emirates; ‡ Department of Electrical and Communication Engineering, College of Engineering, United Arab Emirates University, Al Ain, P.O. Box Abu Dhabi 15551, United Arab Emirates

## Abstract

Spinel-type Zn_2_SnO_4_ and its La- and Ce-modified variants were synthesized via a solid-state route to investigate aliovalent substitution–induced structural, optical, and photoluminescence (PL) modulation. Undoped, La- and Ce-doped Zn_2_SnO_4_ samples were characterized using X-ray diffraction (XRD), Fourier transform infrared (FTIR) spectroscopy, Raman spectroscopy, ultraviolet–visible spectroscopy, photoluminescence, scanning electron microscopy (SEM)–energy-dispersed spectroscopy (EDS), and X-ray photoelectron spectroscopy (XPS) techniques. XRD confirmed the dominant cubic spinel structure (*Fd*3̅*m*:1) with a minor SnO_2_ phase, while rare-earth incorporation induced lattice expansion, microstrain, and reduced crystallite size (32–27 nm). FTIR, Raman, and XPS analyses revealed dopant-induced lattice distortion, enhanced oxygen-vacancy-related defects, and the presence of Zn^2+^, Sn^4+^, La^3+^, and mixed Ce^3+^/Ce^4+^ states. Optical studies showed band gap narrowing from 3.57 eV (undoped) to 3.16 eV (La-doped) and 3.12 eV (Ce-doped), extending visible-light activity. PL results demonstrated dopant-selective color tunability, with orange emission for La-doped and green emission for Ce-doped Zn_2_SnO_4_. These findings establish a clear structure–defect–optical correlation and highlight rare-earth-modified Zn_2_SnO_4_ as a promising material for visible-light optoelectronic, luminescence, and optical sensing applications.

## Graphical abstract

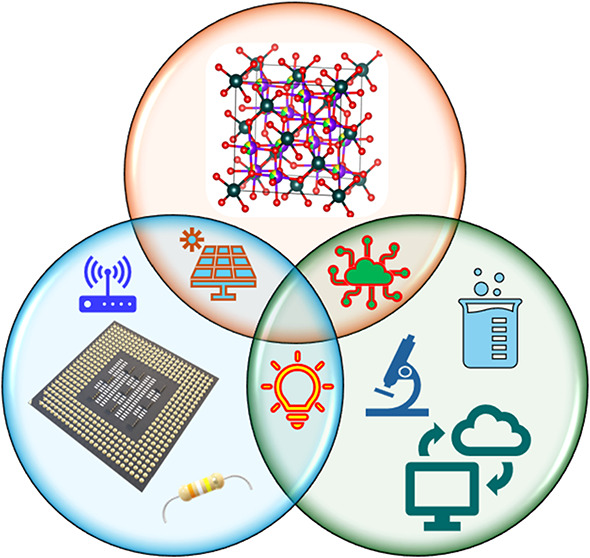

## 1 Introduction

Zinc stannate (Zn_2_SnO_4_) is a cross-cutting wide-band gap spinel oxide that has attracted interest as a single robust material platform for many technological applications.^1–3^ Owing to its inherent thermal and chemical stability, tunable electronic structure, and ease of synthesis, Zn_2_SnO_4_ has been utilized in photocatalytic degradation of organic pollutants under sunlight, sensing gas and volatile organics, as a functional material in photoelectrochemical and photovoltaic devices, and a phosphor/optical material in light-emitting devices.^4^ In photocatalysis, the coexistence of a wide band gap and proper band positions ensures efficient UV absorption and photocatalytic oxidation, and doping or compositing extends light absorption into the visible range. In gas sensing, the synergy between surface oxygen vacancies and crystallite morphology determines analyte adsorption and charge transfer, thus enabling the detection of reducing and oxidizing gases with high sensitivity.^5^ Zn_2_SnO_4_ has also been utilized in heterostructures and electrode architectures for rapid charge transfer in energy storage and conversion devices, suggesting multifunctionality toward integrated catalytic and optoelectronic systems.^6–8^

Defect engineering and targeted doping are two powerful ways to tailor the functional properties of Zn_2_SnO_4_. Aliovalent substitution (e.g., replacement of Sn^4+^ with trivalent rare-earth ions like La^3+^, Ce^3+^/Ce^4+^, and Eu^3+^) creates localized lattice dilation, charge-compensating defects (primarily oxygen vacancies), and new midgap electronic states. These modifications reorganize carrier concentration, trap densities, and recombination channels, thereby directly affecting optical absorption, photoluminescence (PL), and catalytic/sensing kinetics.^9^ Transition-metal impurities (Cu, Fe, Co, Mn, etc.) typically alter the band edges and may add d-state-derived impurity levels that enhance visible-light capture or serve as recombination centers, depending on the chemical environment and concentration.^10–12^ Simultaneously, the dopants are active during sintering and grain growth under thermal treatment: redox-active dopant species or larger dopant ions tend to migrate to grain boundaries, inhibit coarsening, form finer crystallites, and increase the porosity/surface area/active site density for surface-mediated reactions.^13^ Time- and energy-dependent methods, such as time-resolved PL, X-ray photoelectron spectroscopy (XPS), and EPR, are commonly used to determine whether the dopant-induced states are mostly radiative or nonradiative, which is critical for the optimization of photocatalytic versus photonic applications.^14^

Recently, numerous strategies have been developed to enhance the functional performance of Zn_2_SnO_4_ and related AB_2_O_4_ spinel oxides through controlled doping and heterostructure engineering. In Zn_2_SnO_4_, Cu doping has been shown to significantly reduce the optical band gap (down to ∼2.5 eV in hydrothermally synthesized systems) and introduce sub-band states that extend absorption into the visible region, resulting in enhanced photocurrent generation and photocatalytic activity under solar irradiation.^1^ Rare-earth dopants such as Eu^3+^ have also been employed to convert Zn_2_SnO_4_ into an efficient phosphor host, where Eu^3+^ ions occupy well-defined lattice sites and produce narrow orange–red emissions (∼595 to 615 nm) suitable for luminescent devices.^2^ More recently, anion/cation codoping strategies (e.g., N-doped Zn_2_SnO_4_) and Zn_2_SnO_4_-based Z-scheme heterojunctions have been reported to further improve visible-light photocatalysis by combining band gap narrowing with enhanced interfacial charge separation.^15,16^ In addition, Zn_2_SnO_4_ has emerged as a promising electron-transport layer in perovskite solar cells due to its wide band gap (≈3.5–3.8 eV), high electron mobility (≈10–30 cm^2^ V^–1^ s^–1^), and chemical stability, enabling high-efficiency devices and scalable perovskite modules.^17^ Similar defect–property correlations have been reported for other inverse spinel oxides, such as NiFe_2_O_4_ thin films, where oxygen vacancies and cation disorder were shown to strongly tune band gap energy and defect-related photoluminescence.^18^ Moreover, similar defect-assisted optical tuning has been reported in NiFe_2_O_4_–ZrC composites, where lattice strain and phonon coupling induced a band gap redshift and enhanced blue/white photoluminescence.^19^

From a synthesis perspective, Zn_2_SnO_4_ and other AB_2_O_4_ spinels have been prepared by a variety of routes, including hydrothermal or solvothermal methods, sol–gel processes, combustion synthesis, and thin-film deposition techniques such as chemical bath deposition, sputtering, and spray pyrolysis.^20–22^ Within the broader AB_2_O_4_ spinel family, materials such as ZnAl_2_O_4_, ZnGa_2_O_4_, Zn_2_SiO_4_, and ferrite spinels (e.g., NiFe_2_O_4_) have been widely investigated as photocatalysts, phosphors, and functional oxides, exhibiting band gaps spanning approximately 1–8 eV depending on cation chemistry.^23^ ZnAl_2_O_4_ and ZnGa_2_O_4_ are well-known wide-band gap hosts for transition-metal and rare-earth activators, while Zn_2_SiO_4_-based phosphors show strong Eu^3+^- or Co^2+^-activated emission with excellent thermal and chemical stability; ferrite spinels offer narrower band gaps and magnetic functionality but often suffer from increased nonradiative recombination losses.^14,23^ Compared with these systems, Zn_2_SnO_4_ occupies a distinct position as an n-type transparent conducting spinel that uniquely combines optical transparency, high electron mobility, and flexible band-edge alignment.^24,25^ Despite this advantage, most Zn_2_SnO_4_ studies have focused on transition-metal dopants or Eu^3+^ activation, often targeting a single functionality and relying on solution-based synthesis routes. Systematic investigations correlating aliovalent rare-earth substitution, particularly La^3+^ and mixed-valence Ce^3+^/Ce^4+^ with lattice distortion, oxygen-vacancy formation, band gap evolution, and color-tunable defect photoluminescence in bulk Zn_2_SnO_4_ spinels remain limited. Addressing this gap is essential for establishing a comprehensive structure–defect–optical framework for defect-engineered Zn_2_SnO_4_ materials.

The present study advances defect engineering in spinel Zn_2_SnO_4_ by systematically comparing low-level La and Ce substitution (0.3 mol %) at Sn sites using a conventional solid-state reaction route. This dopant level was intentionally selected to probe defect-driven optical modulation while preserving the structural integrity of the cubic spinel lattice. Previous studies on doped AB_2_O_4_ spinels and related oxide semiconductors have shown that low-level aliovalent substitution (<1 mol %) is particularly effective in generating oxygen vacancies and localized defect states that influence band structure and photoluminescence, whereas higher dopant concentrations often promote dopant clustering, secondary phase formation, lattice overdistortion, and nonradiative recombination losses that are especially pronounced in bulk materials synthesized via solid-state routes.^12,26,27^ Accordingly, 0.3 mol % represents an optimized low-doping regime that enables clear identification of substitution-induced lattice expansion, microstrain, oxygen-vacancy formation, band gap narrowing, and dopant-selective visible photoluminescence without introducing competing concentration-dependent effects. By correlating structural (X-ray diffraction (XRD), Raman, Fourier transform infrared (FTIR)), morphological (scanning electron microscopy (SEM)/energy-dispersed spectroscopy (EDS)), and optical–electronic (UV–Vis, PL, CIE) signatures, this work establishes a coherent structure–defect–optical framework and demonstrates dopant-dependent color tuning (violet → yellowish orange → green) in Zn_2_SnO_4_. This combined structural–optical–morphological mapping elucidates material design principles for multifunctional Zn_2_SnO_4_ platforms (sensing, photocatalysis, and phosphors), and it defines controllable route-level parameters (doping level and sintering profile) to optimize functionality for targeted applications.

## 2 Experimental Section

### 2.1 Materials and Synthesis

Zinc oxide (ZnO), tin­(IV) oxide (SnO_2_), lanthanum­(III) oxide (La_2_O_3_), and cerium­(IV) oxide (CeO_2_), all of analytical grade (99.9% pure, Sigma-Aldrich), were used as precursors. Polycrystalline Zn_2_SnO_4_ and its La- and Ce-doped analogues (0.3 mol % doping at the Sn site) were synthesized using standard solid-state reactions. ZnO, SnO_2_, La_2_O_3_, and CeO_2_ were combined in the desired proportions and manually ground in an agate mortar to obtain a homogeneous mixture.

The homogenized powders were initially calcined in an alumina crucible at 900 °C for 12 h. After cooling, the calcined product was milled and subjected to a secondary sintering treatment at 1100 °C for 6 h, with intermittent grinding to enhance phase purity. The resulting fine powders were pressed into pellets 10 mm in diameter and then sintered in air at 1200 °C for 2 h. Controlled cooling to room temperature was maintained at a rate of 5 °C min^–1^ between successive stages of heat treatment, in order to achieve uniform microstructural growth and compositional homogeneity. Figure 1. illustrates the schematic of the procedure for preparing undoped, La and Ce-doped Zn_2_SnO_4_ via the conventional solid-state reaction route.

**Figure 1:**
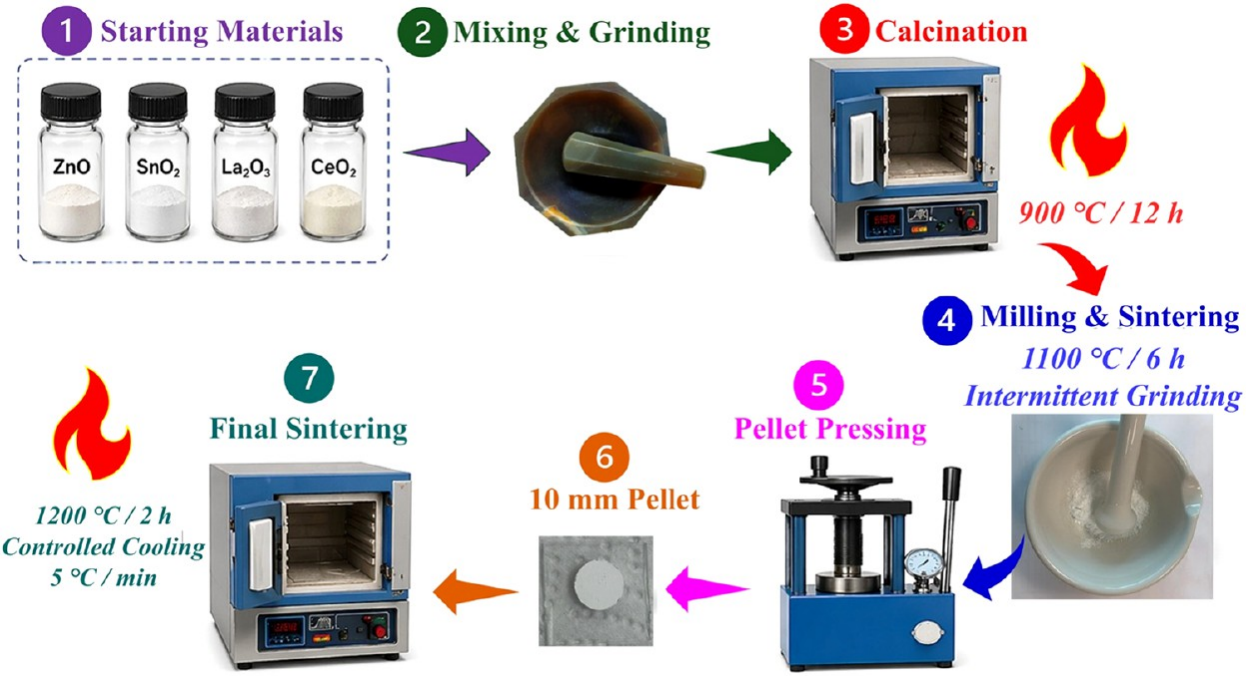
Schematic of the procedure for preparing undoped and La- and Ce-doped Zn_2_SnO_4_ via the conventional solid-state reaction route.

### 2.2 Characterization Techniques

All synthesized samples were characterized at room temperature under ambient laboratory conditions. The phase purity and crystallographic structure were examined using a Shimadzu LabX XRD-6100 diffractometer equipped with Cu Kα radiation (λ = 1.5406 Å). Diffraction patterns were recorded over a 2θ range of 10–80° with a step size of 0.02° and a scan rate of 2° min^–1^. The angular accuracy of the instrument is ±0.02°. Phase identification was performed using the ICDD PDF-4 database. Detailed structural parameters, including lattice constants, site occupancies, and isotropic displacement parameters, were obtained through full-profile Rietveld refinement using the FULLPROF software package.^28^ The quality of refinement was evaluated using *R*_p_, *R*_wp_, and χ^2^ values. Fourier transform infrared (FTIR) spectra were recorded using a VARIAN 3100 FTIR spectrometer (Excalibur series) equipped with Opus 6.5 software for data acquisition and analysis. The measurements were carried out in the range of 400–4000 cm^–1^ with a spectral resolution of 4 cm^–1^. Raman measurements were performed on a Horiba Jobin-Yvon HR 800 spectrometer using a 633 nm He–Ne laser as the excitation source, with an approximate spectral resolution of 1 cm^–1^. Raman peaks were analyzed using Lorentzian fitting functions to extract peak positions and full width at half-maximum (FWHM) values for evaluating lattice distortion and phonon broadening. Surface morphology was examined using a JEOL JSM-6010PLUS scanning electron microscope (SEM) operated at an accelerating voltage of 15 kV. Elemental composition and mapping were carried out using an attached energy-dispersive X-ray spectroscopy (EDS) system. X-ray photoelectron spectroscopy (XPS) measurements were conducted using a Physical Electronics PHI 5000 VersaProbe III system with monochromatic Al Kα radiation (*h*ν = 1486.6 eV). The energy resolution was approximately ±0.5 eV, and binding energies were calibrated using the C 1s peak at 284.8 eV as a reference. High-resolution spectra were deconvoluted using CASA-XPS software with Gaussian–Lorentzian peak fitting and Shirley background subtraction. Optical absorption measurements were performed using a JASCO V-670 UV–Vis spectrophotometer in the wavelength range of 200–1000 nm, with a wavelength accuracy of ±0.5 nm. Optical band gaps were determined from Tauc plots assuming a direct allowed transition. Photoluminescence (PL) spectra were recorded using a Shimadzu RF-5301PC spectrofluorophotometer over the 200–900 nm range. Excitation wavelengths were selected individually for each sample based on their absorption characteristics to effectively probe the dominant electronic transitions, while instrumental parameters such as slit widths and scan conditions were kept constant to ensure consistency across measurements.

## 3 Results and Discussion

### 3.1 XRD Analysis

Rietveld refinement patterns of synthesized Zn_2_SnO_4_, Zn_2_SnO_4_:0.3La, and Zn_2_SnO_4_:0.3Ce are shown in Figure 2. The pattern of undoped Zn_2_SnO_4_ strongly suggests a single dominant cubic spinel phase with space group *Fd*3̅*m*:1, which is consistent with JCPDS Card No. 24-1470.^25^ The major peaks at 2θ = 17.8, 29.2, 34.4, 35.9, 41.7, 51.6, 56.4, and 62.7° correspond to the (111), (220), (311), (222), (400), (422), (511), and (440) planes, respectively. However, several weak reflections at 2θ ≈ 26.6, 33.8, and 51.8° are observed, consistent with tetragonal SnO_2_ with a cassiterite structure (JCPDS Card No. 41-1445).^12^ No distinct rare-earth oxide phases are observed, suggesting that La^3+^ and Ce^3+^/Ce^4+^ ions are incorporated into the Zn_2_SnO_4_ matrix at low substitution levels. Weak reflections corresponding to SnO_2_ are also present in all samples, with slightly increased intensity upon doping. This is consistent with previous reports of Zn_2_SnO_4_/SnO_2_ heterostructures, where minor SnO_2_ coexists with the spinel phase due to slight off-stoichiometry or local tin segregation during high-temperature synthesis.^29–31^ Substitution of larger La^3+^ (1.16 Å) and Ce^3+^ (1.14 Å) for smaller Sn^4+^ (0.69 Å) can disrupt cation ordering and diffusion kinetics during solid-state synthesis (1100 °C/6 h), facilitating the formation of trace SnO_2_ upon cooling. The estimated amount of this secondary phase remains low (4–6%), indicating the formation of a quasi-homogeneous solid solution rather than significant phase separation. Reports on defect-rich Zn_2_SnO_4–*x*_/SnO_2–*x*_ systems further show that such minor SnO_2_ phases typically do not dominate the structural framework of Zn_2_SnO_4_ but can coexist without compromising the primary spinel lattice. Importantly, the dominance of the Zn_2_SnO_4_ phase and the absence of detectable rare-earth oxide impurities confirm effective low-level substitution and justify focusing subsequent optical and electronic analyses on the defect-modified Zn_2_SnO_4_ host.

**Figure 2:**
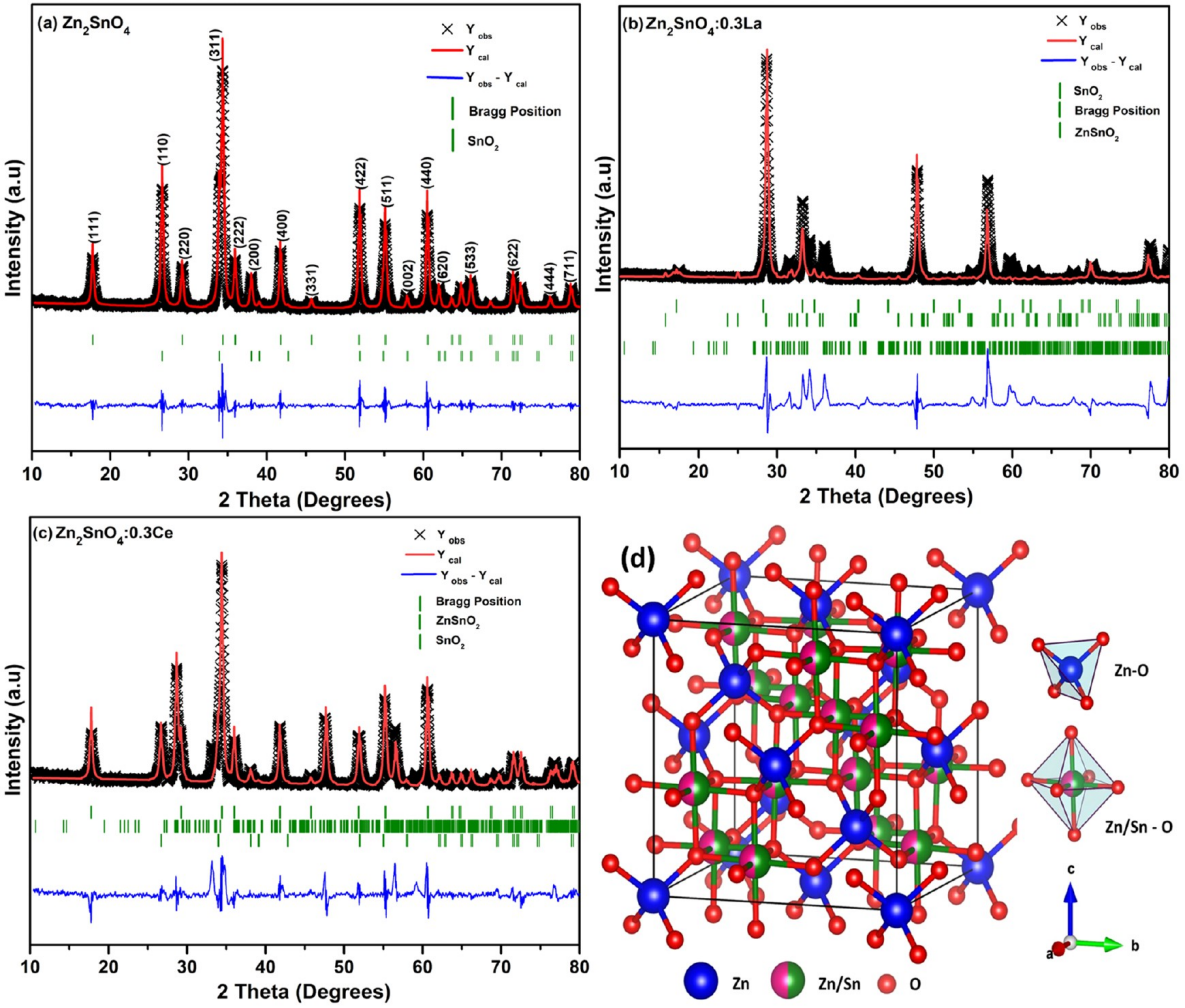
Rietveld refinement patterns of (a) Zn_2_SnO_4_, (b) Zn_2_SnO_4_:0.3La, (c) Zn_2_SnO_4_:0.3Ce, and (d) crystal structure of Zn_2_SnO_4_ spinel-type nanopowders.

A careful comparison of the diffraction patterns revealed a systematic shift of the (311) peak toward lower 2θ values with doping from 34.42° in undoped Zn_2_SnO_4_ to 34.29 and 34.26° in La- and Ce-doped samples, respectively. This shift confirms lattice expansion upon substitution of dopants with larger ionic radii at the Sn sites. The lattice parameter *a* derived using the (311) reflection is 8.637 Å for Zn_2_SnO_4_, 8.940 Å for Zn_2_SnO_4_:0.3La, and 8.652 Å for Zn_2_SnO_4_:0.3Ce. The lattice expansion percentages (Δ*a*%) are +0.27% for La doping and +0.34% for Ce doping, signifying effective structural alteration by substitutional inclusion.

The average crystallite size (*D*) was estimated using the Debye–Scherrer equation^26^

1 $$D=\frac{K\lambda }{\beta \cos\theta }$$

where *K* = 0.9, λ = 1.5406 Å (Cu Kα), β is the corrected full width at half-maximum (FWHM) in radians, and θ is the Bragg angle. After correction for instrumental broadening, the average *D* values of the strongest reflections are 32 nm for Zn_2_SnO_4_, 28 nm for Zn_2_La_0.3_Sn_0.7_O_4_, and 27 nm for Zn_2_Ce_0.3_Sn_0.7_O_4_. The reduction in crystallite size indicates that the addition of La and Ce increases the lattice strain and defect density, hindering the coalescence of grains during sintering.^27^ In Figure 3a–c shows the Williamson–Hall (W–H) analysis, which indicates the presence of microstrain (ε ≈ 1.4 × 10^–3^–3.6 × 10^–3^), suggesting localized lattice distortion due to dopant addition.

**Figure 3:**
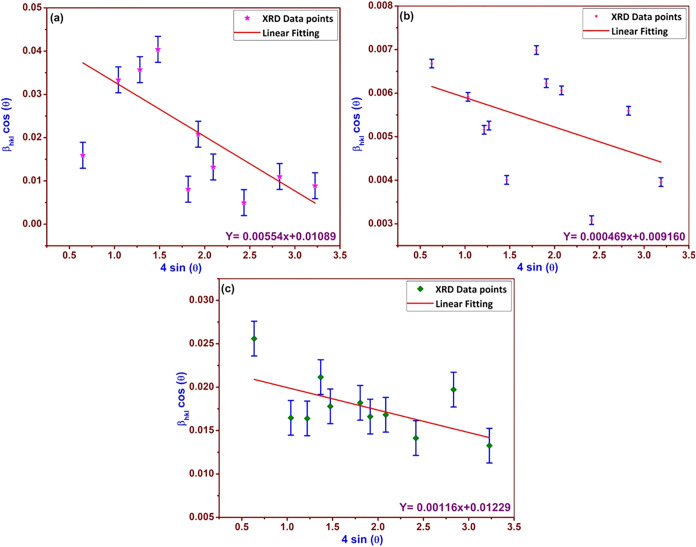
W–H plot of (a) undoped, (b) La-doped, and (c) Ce-doped Zn_2_SnO_4_ spinel-type nanopowders. Error bars indicate ± standard deviation of five measurements.

The refined crystallographic parameters (Table 1) and atomic coordinates with isotropic displacement parameters (Table 2) further corroborate the successful substitutional incorporation of rare-earth ions and the presence of an oxygen-defect-mediated lattice modification. Overall, XRD analysis confirms that doping with La and Ce causes minor lattice expansion, increased microstrain, and reduced crystallite size but maintains the cubic spinel Zn_2_SnO_4_ structure, reflecting the successful incorporation of dopant ions into the host lattice through substitutional mechanisms.

**Table 1:** Refined Crystallographic Parameters of Zn_2_SnO_4_, Zn_2_SnO_4_:0.3Ce, and Zn_2_SnO_4_:0.3La Obtained from Rietveld Refinement

Parameter	Zn_2_SnO_4_	Zn_2_SnO_4_:0.3Ce	Zn_2_SnO_4_:0.3La
Crystal system	cubic	cubic	cubic
Space group	*Fd*3̅*m*:1	*Fd*3̅*m*:1	*Fd*3̅*m*:1
Lattice parameter *a* (Å)	8.6378 ± 0.0005	8.6525 ± 0.0006	8.9404 ± 0.0008
α = β = γ (°)	90	90	90
Unit cell volume V (Å^3^)	644.48 ± 0.08	647.77 ± 0.09	714.61 ± 0.12
*R*_p_ (%)	7.40	11.59	15.04
*R*_wp_ (%)	10.87	15.72	17.95
χ^2^	0.72	1.32	3.79

**Table 2:** Atomic Coordinates and Isotropic Displacement Parameters of Zn_2_SnO_4_, Zn_2_SnO_4_:0.3Ce, Zn_2_SnO_4_:0.3La

Atom	*x*	*y*	*z*	Occ.	*U*_eq_ (Å^2^)
Zn_2_SnO_4_					
Zn1	0.00000	0.00000	0.00000	1	0.00164
Zn2	0.62500	0.62500	0.62500	0.5	0.00306
Sn2	0.62500	0.62500	0.62500	0.5	0.00306
O	0.38138	0.38138	0.38138	1	0.0024
Zn_2_SnO_4_:0.3Ce					
Zn1	0.00000	0.00000	0.00000	1	0.009900
Zn2	0.62500	0.62500	0.62500	0.5	0.009900
Sn2	0.62500	0.62500	0.62500	0.35	0.009900
Ce2	0.62500	0.62500	0.62500	0.15	0.009900
O	0.39000	0.39000	0.39000	1	0.009900
Zn_2_SnO_4_:0.3La					
Zn1	0.00000	0.00000	0.00000	1	0.34178
Zn2	0.62500	0.62500	0.62500	0.5	0.34178
Sn2	0.62500	0.62500	0.62500	0.35	0.34178
La2	0.62500	0.62500	0.62500	0.15	0.34178
O	0.3900	0.3900	0.3900	1	0.34178

### 3.2 FTIR Spectroscopy

FTIR spectra of undoped and doped Zn_2_SnO_4_ were recorded to study the functional groups, nature of bonding, and lattice vibrations associated with the spinel structure. The spectra of all samples (Figure 4) show characteristic absorption bands corresponding to metal–oxygen vibrations, hydroxyls, and adsorbed water molecules.^32^

**Figure 4:**
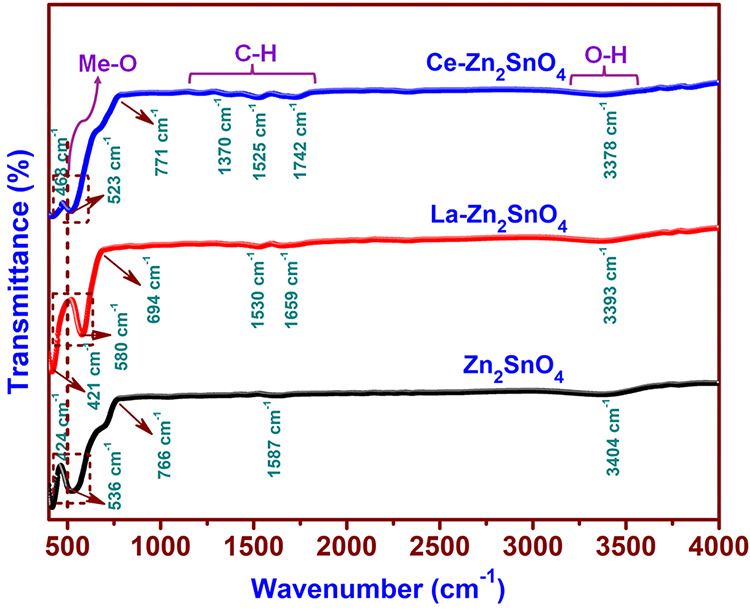
FTIR spectra of undoped, La-doped, and Ce-doped Zn_2_SnO_4_ spinel-type nanopowders.

The broad and strong absorption band within 3425–3435 cm^–1^ is attributed to O–H stretching vibrations of adsorbed hydroxyls or water molecules on the surface.^33^ This band indicates the hydrophilic nature of the oxide surface and water absorption during grinding and synthesis, which is common in powders synthesized via solid-state reactions.^34^ A less intense band near 1620–1635 cm^–1^ and a broad band within 3425–3435 cm^–1^ are assigned to the H–O–H bending vibration and the hydroxyl or water species, respectively, of adsorbed water.^35^

In the lower wavenumber region (400–800 cm^–1^), there are sharp bands corresponding to vibrations of metal–oxygen in the spinel lattice.^36^ The band at 540–555 cm^–1^ is assigned to Sn–O stretching vibrations, and that at 420–430 cm^–1^ to Zn–O vibrations at tetrahedral sites of the spinel lattice. These assignments are consistent with the literature on Zn_2_SnO_4_ inverse spinel phases. The absorption peaks confirm the formation of a metal–oxygen network and effective crystallization of the cubic spinel phase, in agreement with the XRD data.^37^ Interestingly, these metal–oxygen stretching bands in the doped samples shift slightly to lower wavenumbers (Δν ≈ 5–10 cm^–1^) due to substitution of larger La^3+^ and Ce^3+^ ions at the Sn^4+^ sites. This substitution causes minor lattice distortion and lengthening of metal–oxygen bonds, reducing the vibrational frequency as generally observed in rare-earth-doped spinel oxides.^38^ No additional impurity peaks of rare earth oxide were observed, implying that La^3+^ and Ce^3+^ were mainly introduced into the Zn_2_SnO_4_ structure rather than segregating into a secondary phase, in agreement with the XRD analysis.

The lack of significant bands within 1000–1500 cm^–1^ indicates the absence of residual nitrate or carbonate from the precursor materials, confirming complete solid-state reaction and high purity of the resulting powders.^39^ The overall FTIR spectral features, from hydroxyl-related bands to typical Zn–O and Sn–O vibrations, reveal that the spinel structure remains intact upon doping with rare earth elements despite minor shifts of the bands due to structural changes after ion substitution. In conclusion, FTIR analysis confirms the successful formation of Zn_2_SnO_4_ spinel phase and provides evidence of lattice distortion due to La and Ce incorporation. The unique metal–oxygen vibrations and adsorbed hydroxyl groups highlight the surface chemistry and structural integrity of the synthesized materials. These observations, combined with XRD and crystallite size data, provide comprehensive information about the microstructural and structural characteristics of undoped and doped Zn_2_SnO_4_ samples.

### 3.3 UV–Vis Absorption and Optical Band Gap Analysis

Optical absorption characteristics of undoped and doped Zn_2_SnO_4_ were investigated through UV–vis spectroscopy in the 200–1000 nm wavelength range, and the optical band gaps were determined using Tauc plots.^40^ In their absorbance spectra (Figure 5, top panels), all three samples exhibit a sharp absorption edge near the UV region, which is characteristic of wide-band gap semiconductors. Undoped Zn_2_SnO_4_ shows an intrinsic absorption onset at around 347 nm, which is typical of strong interband valence-to-conduction band transitions.^41^ After doping with La^3+^ and Ce^3+^, the absorption edge shifts smoothly to longer wavelengths, with cut-offs at approximately 393 and 397 nm, respectively. This redshift of the absorption edge indicates an enhanced light-harvesting ability and a reduction in the optical band gap upon rare earth substitution.^42^

**Figure 5:**
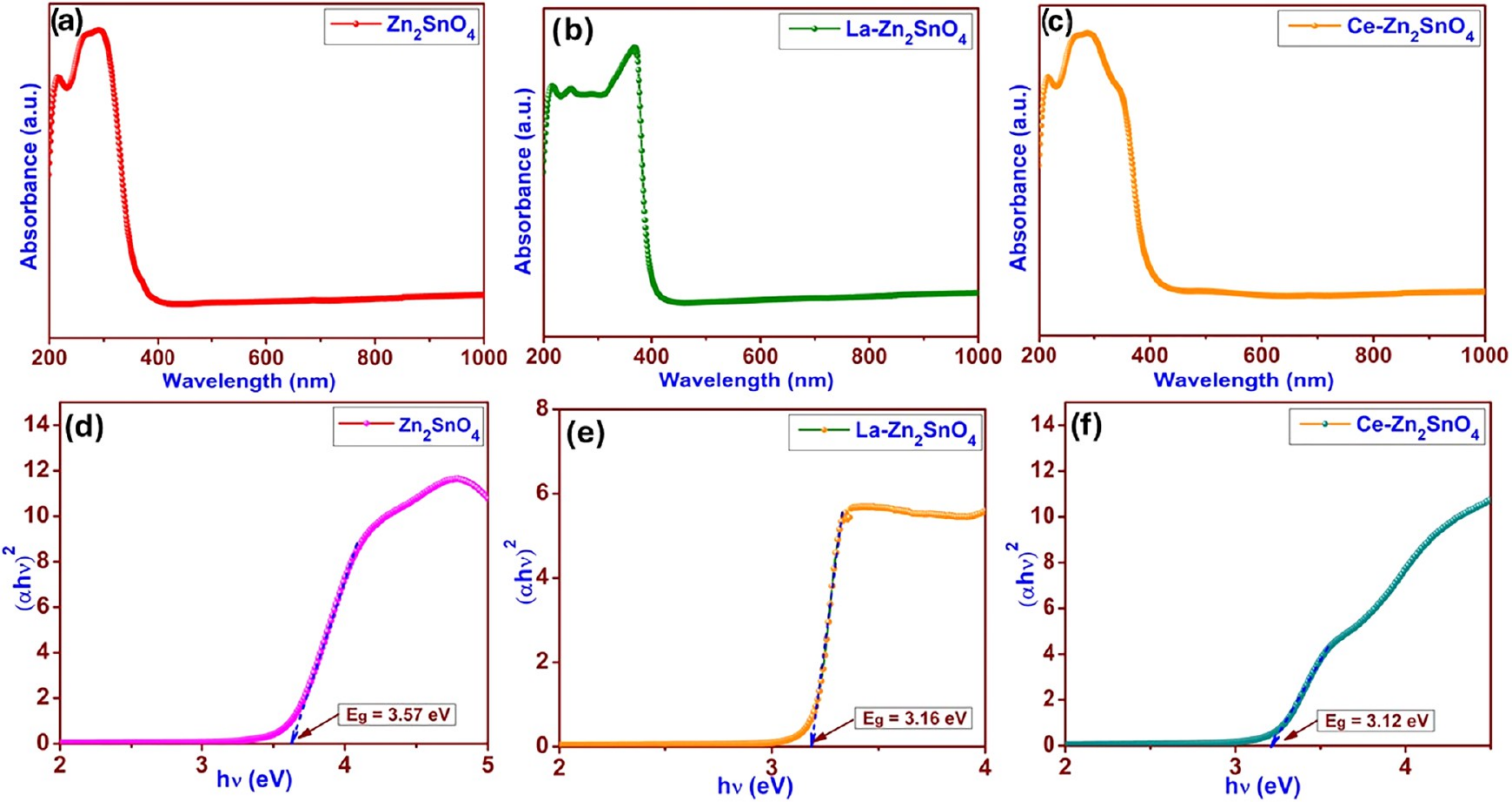
(a–c) UV–vis absorption spectra and (d–f) corresponding optical band gap plots derived from Tauc’s relation for (a, d) undoped, (b, e) La-doped, and (c, f) Ce-doped Zn_2_SnO_4_ spinel-type nanopowders.

Tauc plots of the absorption data ((α*h*ν)^2^ vs *h*ν (eV), bottom panels in Figure 5) were used to estimate the optical band gap (*E*_g_) by extrapolating the linear portion of the curves to the photon energy axis. The direct optical band gap of pristine Zn_2_SnO_4_ (3.57 eV) is in reasonable agreement with the reported values for stoichiometric Zn_2_SnO_4_ spinel.^43^ The band gap energy decreases significantly to 3.16 and 3.12 eV for La-doped and Ce-doped Zn_2_SnO_4_, respectively. This change can be attributed to lattice distortion and formation of localized impurity states in the band structure because of the substitution of La^3+^ and Ce^3+^ ions at Sn^4+^ sites. Such a substitution disrupts the electronic environment, reduces orbital overlaps, and creates tail states near the edges of the conduction or valence bands, thereby lowering the transition energy.

The redshift of the absorption edge and the corresponding band gap reduction indicate that rare-earth doping effectively modifies the electronic structure of Zn_2_SnO_4_, without affecting its semiconducting nature.^44^ These changes enhance the optical response in the near-UV-to-visible regime, which is desirable for optoelectronic and photocatalytic applications where efficient light absorption and a reduced band gap are necessary for enhanced performance.^45^

### 3.4 Raman Spectroscopy

The Raman spectra of undoped and doped Zn_2_SnO_4_ samples (Figure 6) provide valuable information on the local structural ordering, lattice vibrations, and vibrational dynamics. Zn_2_SnO_4_ crystallizes in the normal cubic spinel structure with space group *Fd*3̅*m*:1 (No. 227),^46^ in which Zn^2+^ is located at the tetrahedral (A) sites and Sn^4+^ at the octahedral (B) sites with coordinated oxygen. Group theory predicts the zone-center optical phonon modes to be Γ_Raman_ = A_1g_ + E_g_ + 3F_2g_ (Raman active) + 5F_1u_ (IR active) + 2A_2u_ + 2E_u_ + 2F_2u_ (silent).^47^ The Raman-active modes A_1g_, Eg, and F_2g_ are due to various vibrations of cation and oxygen sublattices at the octahedral and tetrahedral sites, arising from oxygen motions that are synchronized with the two sublattices of the cations. The high-energy A_1g_ mode is primarily due to symmetric O stretching within the tetrahedral (A) units (M_A_–O), the E_g_ mode arises from symmetric bending/oxygen deformation in coupled tetrahedra and octahedra, and the three F_2g_ modes from lower-energy vibrational/translation and bending motions involving both A- and B-site cations and their coordinating oxygens. First-order Raman scattering serves as a sensitive probe for (i) cation site occupation and mass/force constant changes, (ii) local strain and disorder, and (iii) defect/impurity modes or second-order features.^48^

**Figure 6:**
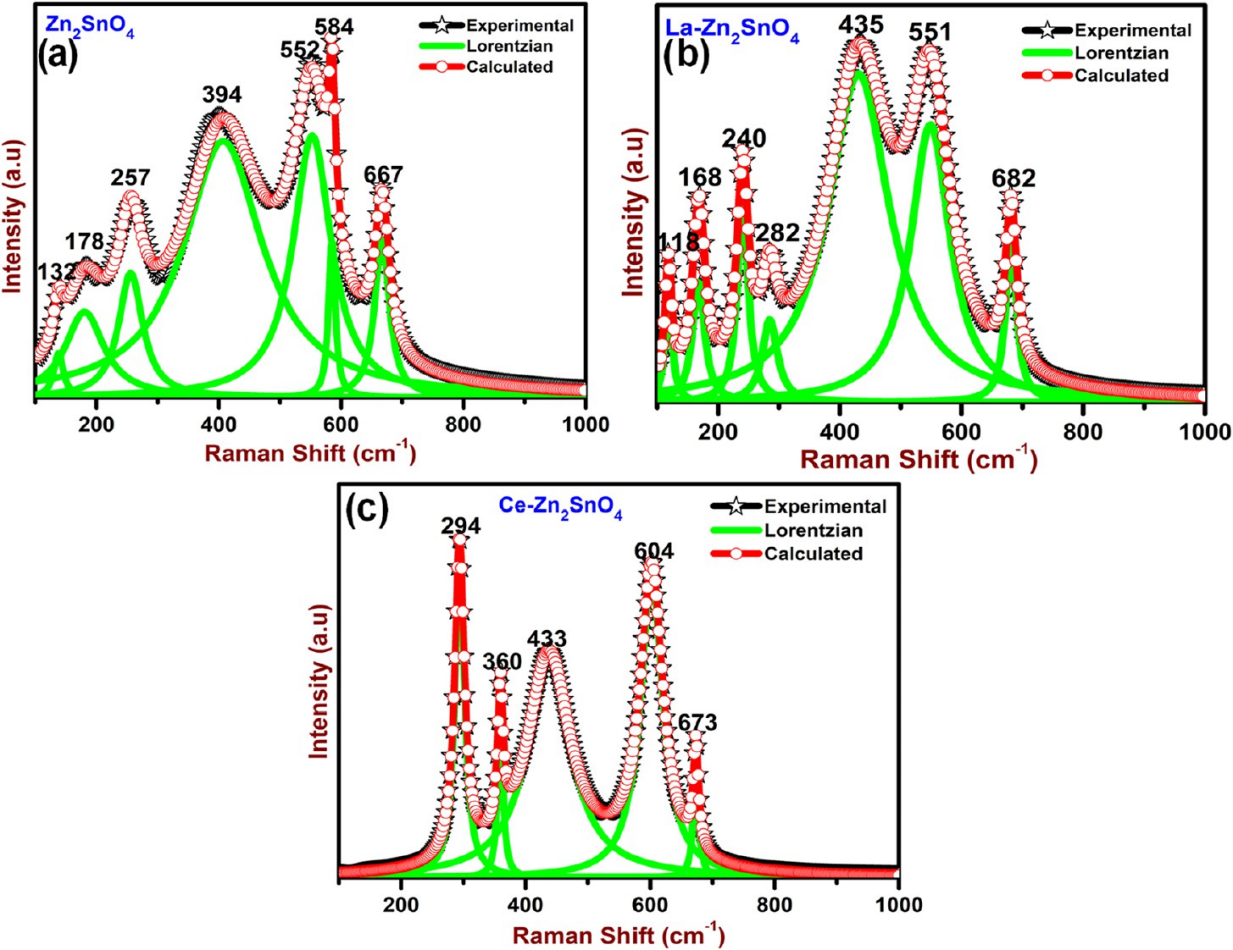
Raman spectra and Lorentzian fitting curves for (a) undoped, (b) La-doped, and (c) Ce-doped Zn_2_SnO_4_ spinel-type nanopowders.

In this study, the Raman spectrum of undoped Zn_2_SnO_4_ consists of sharp peaks at approximately 137, 178, 257, 394, 552, 584, and 667 cm^–1^. The wide band near 584 cm^–1^ is associated with A_1g_ symmetric stretch of SnO_6_ octahedra, and those at 394 and 552 cm^–1^ are assigned to F_2g_ modes of asymmetric stretching and bending vibrations of Sn–O bonds. The translational and vibrational movements of Zn^2+^ and Sn^4+^ and the collective vibration of the oxygen framework are responsible for the three low-frequency modes at 137, 178, and 257 cm^–1^. The feature near 667 cm^–1^ is attributed to second-order scattering or distortion-activated phonon, showing small losses under ideal crystallinity.^49^

The incorporation of La^3+^ and Ce^3+^ into the Zn_2_SnO_4_ lattice significantly influences the vibrational environment, creating subtle structural distortions and changing the local bonding structure. Raman bands at 168, 240, 282, 435, 551, and 682 cm^–1^ were observed in the spectrum of La-doped Zn_2_SnO_4_. The high-intensity peak near 435 cm^–1^ indicates a change in the F_2_g mode, which may be related to lattice strain as well as changing Sn–O bond strength because the larger La^3+^ ion replaces smaller ones. The new modes at 168 and 240 cm^–1^ reflect higher disorder and possible activation of silent modes because of relaxed symmetry. The redshift and broadening of the A_1g_ mode at approximately 551 cm^–1^ also support defect-induced phonon softening. The Ce-doped sample exhibits Raman modes at approximately 294, 360, 433, 604, and 673 cm^–1^. The normal A_1g_ stretching band is displaced to 604 cm^–1^, indicating changes in the SnO_6_ octahedral geometry. The 433 cm^–1^ mode is assigned to F_2g_ vibration, and the low-frequency top near 294 cm^–1^ is due to coupled cationic motion with simultaneous lattice deformation. The additional band at 673 cm^–1^ again points toward defect-activated phonon modes due to lattice distortion and oxygen vacancy formation.^50^

The progressive widening, broadening, and intensity modulation of Raman peaks upon doping with rare earths indicate increased lattice disorder, local strain, and defect state formation within the Zn_2_SnO_4_ spinel matrix.^51^ These are due to the mismatch of ionic radii and local charge imbalance when La^3+^ and Ce^3+^ substitute Sn^4+^, leading to phonon softening and activation of additional vibrational modes. These changes can critically influence the charge transport, electron–phonon coupling, and general functional properties in a way desirable for optoelectronic and catalytic applications.^52^ In summary, our Raman results indicate successful incorporation of La and Ce into the host lattice and provide strong evidence of structural distortion and phonon modulation without altering the fundamental spinel framework. The overall Raman-active phonon modes of undoped, La and Ce-doped Zn_2_SnO_4_ spinels obtained from Lorentzian fitting of normalized Raman spectra are listed in Table 3.

**Table 3:** Raman-Active Phonon Modes of Undoped, La-Doped, and Ce-Doped Zn_2_SnO_4_ Spinels Obtained from Lorentzian Fitting of Normalized Raman Spectra

Sample	Mode assignment	Symmetry	Peak position (cm^–1^)	FWHM (cm^–1^)	Physical significance
Zn_2_SnO_4_	Sn–O symmetric stretching	A_1g_	584	19	well-ordered spinel lattice
	O bending/lattice vibration	F_2g_	394	23	stable octahedral coordination
	translational mode	F_2g_	257	21	low lattice disorder
Zn_2_SnO_4_:0.3La	Sn–O symmetric stretching	A_1g_	551	26	lattice distortion due to La^3+^ substitution
	O bending/defect-related mode	F_2g_	435	29	increased phonon broadening from defects
	translational/disorder mode	F_2_g	240	31	enhanced lattice strain
Zn_2_SnO_4_:0.3Ce	Sn–O symmetric stretching	A_1g_	604	33	strong local distortion and defect coupling
	O bending/vacancy-related mode	F_2g_	433	35	oxygen-vacancy-induced disorder
	translational/defect mode	F_2g_	294	37	highest lattice disorder among samples

### 3.5 Surface Morphology and Elemental Analysis

The SEM micrograph of undoped Zn_2_SnO_4_ (Figure 7a) shows highly aggregated, irregularly shaped clusters formed by the sintering of primary particles. At low magnification, the material appears as a dense micron-sized agglomerate, but at higher magnification, each agglomerated particle can be observed to be composed of much finer subunits with a rough, granular surface. This hierarchical structure is typical of materials prepared by high-temperature solid-state processes with intermediate and final grinding and long thermal residence times (900 °C × 12 h and 1100 °C × 6 h). Specifically, multiple cycles of sintering and particle coalescence produce fused clusters, with an exterior morphology defined by necking between the original crystallites.^53^ The relatively narrow XRD peaks and the Scherrer crystallite size of ∼32 nm, also support this observation, i.e., the dominant crystallites are nanoscale while the visible SEM structures are micron-scale agglomerates of those nanocrystals.^54^

**Figure 7:**
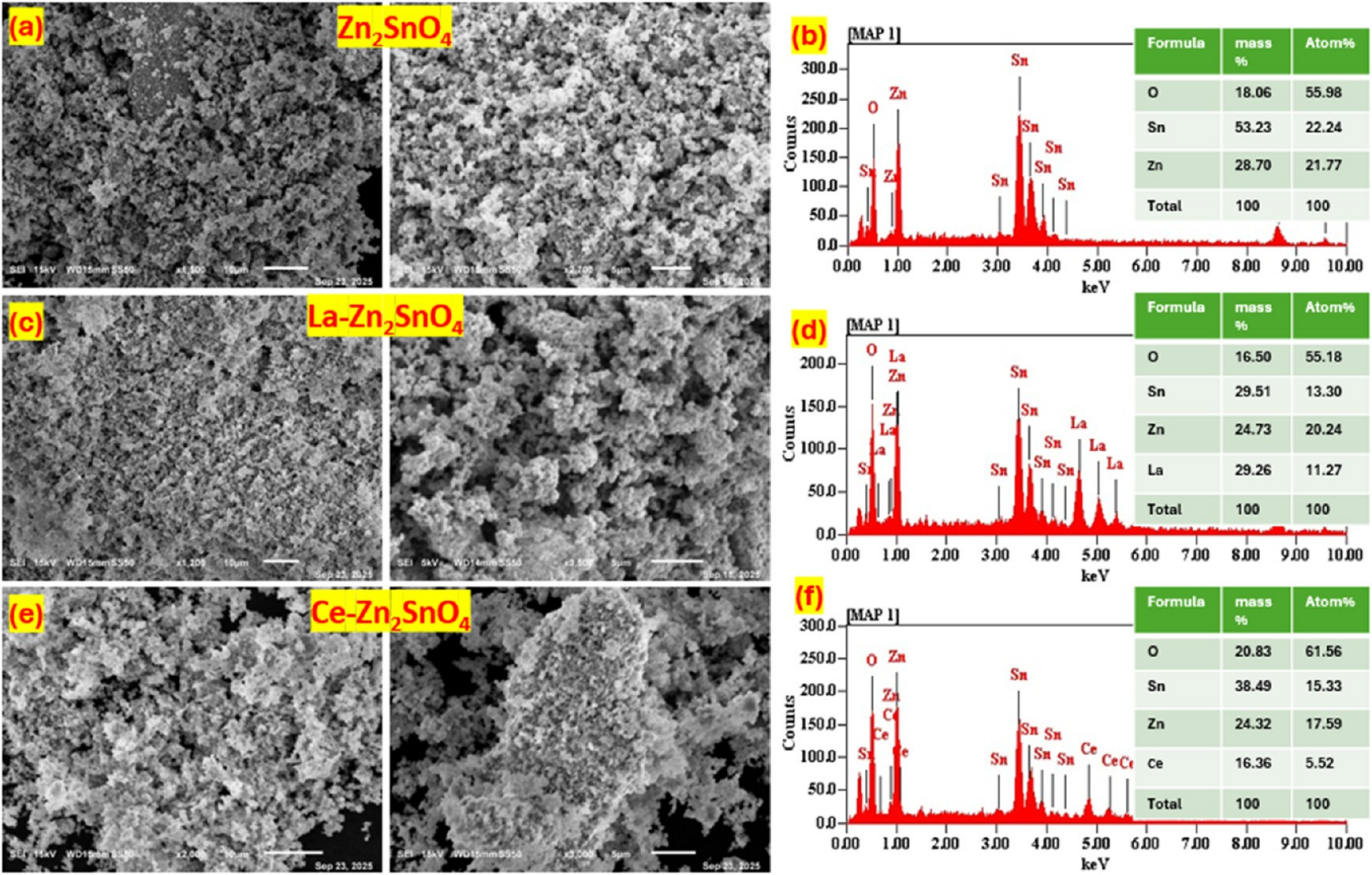
(a, c, e) SEM micrographs and (b, d, f) corresponding EDS spectra of (a, b) undoped, (c, d) La-doped, and (e, f) Ce-doped Zn_2_SnO_4_ spinel-type nanopowders.

The La-doped Zn_2_SnO_4_ has a visibly different surface structure. Compared to the undoped sample, there are more open, sponge-like clusters with finer, more uniform granules at higher magnification (Figure 7c). The particles are less sintered together in the solid plates and have larger interparticle pores.^55^ This morphology is consistent with the smaller average Scherrer crystallite size of ∼28 nm, indicating that La incorporation suppresses grain coarsening during the high-temperature process through (1) segregation at grain boundaries and (2) lattice strain that slows mass transport. A more porous surface and a larger number of small subunits increase the accessible surface area, which is desirable for surface-sensitive applications such as adsorption and photocatalysis.^56^

The Ce-doped sample displays a unique morphology (Figure 7e). There are somewhat coarser but still extremely textured aggregates with an irregular surface, together with regions of relatively high-density nodular growth. Compared with the La-doped sample, this sample contains regions with slightly denser apparent packing but retains much of the nanogranular character of the principal domains. It has the lowest calculated Scherrer size (∼27 nm) among the three samples, indicating that Ce substitution causes the most extensive crystallographic disorder and the most pronounced inhibition of crystal growth.^57^ The presence of mixed Ce^3+^/Ce^4+^ chemistry (which is native to Ce) and associated oxygen vacancy creation probably promotes local defect clustering and modifies the sintering properties, leading to the observed mixture of dense and porous microstructures.

EDS analysis confirmed the elemental compositions of each sample. All three samples showed only the expected elements O, Sn, Zn, and the target rare-earth dopants with no sign of contaminants in the areas examined in Figure 7b,d,f. Using the representative spectra, the atomic percentages (atom %) were calculated as follows: undoped Zn_2_SnO_4_: O 55.98 atom %, Sn 22.24 atom %, Zn 21.77 atom %; La-doped Zn_2_SnO_4_: O 55.18 atom %, Sn 13.30 atom %, Zn 20.24 atom %, La 11.27 atom %; and Ce-doped Zn_2_SnO_4_: O 61.56 atom %, Sn 15.33 atom %, Zn 17.59 atom %, Ce 5.52 atom %. These results confirm the successful incorporation of La and Ce into the matrix and identify oxygen as the dominant surface species. Because EDS is a surface-sensitive, semiquantitative technique, the measured atomic percentages deviate slightly from the stoichiometry based on nominal formulas, with excess Sn (measured at 22.24 vs 14.29 atom % in theory) and deficient Zn (21.77 vs 28.57 atom %). In the La-doped sample, the measured La content is also higher than the theoretical one (11.27 vs 4.29 atom %). In the Ce-doped sample, the Ce content is measured to be 5.52 atom %, whereas the theoretical content is 4.29 atom %. The higher-than-expected rare-earth contents are likely due to an interplay among surface enrichment, local inhomogeneity (micrometer-scale concentration of the dopant), preferred X-ray yield of heavy elements, and the known limitations of EDS quantification (ZAF matrix effects, absorption, and fluorescence).^58^ EDS can determine the presence and distribution of elements, but does not always yield the correct bulk concentrations.

Also, EDS cannot distinguish the crystallographic phases made of isomorphic elements. Therefore, we unraveled the faint SnO_2_ XRD reflections of all samples, because SnO_2_ consists of the same major elements as the Zn–Sn–O matrix, and therefore its presence may not be evident in the EDS spectra. The higher relative Sn percentage determined by EDS in the undoped sample (22.24 atom %) and the observation that Sn-rich zones are unaffected are thus consistent with the XRD detection of minor SnO_2_ phase. As complementary techniques, XRD picks up the trace SnO_2_ phase crystallographically, and EDS shows surface Sn enrichment that is consistent with secondary phase and/or surface segregation of Sn.

The elemental compositions obtained from EDS show higher apparent La and Ce contents than the nominal dopant concentrations. This discrepancy arises from the semiquantitative and surface-sensitive nature of EDS, particularly for heavy rare-earth elements, where matrix effects, X-ray absorption, and fluorescence can lead to an overestimation of dopant concentrations. In addition, local surface enrichment and microscale compositional heterogeneity can further influence the measured values. Therefore, EDS is used in the present study primarily to confirm the presence and spatial distribution of La and Ce dopants rather than for precise bulk compositional quantification. The nominal dopant concentration remains defined by the precursor stoichiometry employed during the synthesis.

The morphological and structural results can be understood in the context of the solid-state synthesis process. Mechanical mixing of solids allows intimate contact between the reactants and leads to homogeneous nucleation, whereas high-temperature final sintering promotes crystallite growth and necking. As a result, micrometer-sized agglomerates of nanoscale primary crystals were observed in all samples. Dopants with larger cationic radii (La and Ce) and different valences from Sn alter the rates of cation diffusion and tend to segregate at the grain boundaries. This grain boundary segregation retards grain coalescence (hence, a smaller Scherrer *D* value) and increases the porosity in the La-doped sample. The redox-active Ce species enhances the development of defects (oxygen vacancies) that affect coarsening and local densification behavior.^59^ From an application perspective, the reduced crystallite size, increased surface area, and higher porosity of the doped samples are advantageous for catalytic, photocatalytic, and sensing applications.

The elemental mapping results confirm the synthesis and chemical purity of undoped Zn_2_SnO_4_ (Figure 8a) and successful doping with La (Figure 8b) and Ce (Figure 8c). All three samples consist of agglomerated fine particles. Most importantly, the distributions of Sn (yellow), O (red), and La/Ce (pink) are even and match the SEM morphology, confirming the stannate-phase structure of the host. Furthermore, the doped La and Ce atoms overlap evenly with the Zn, Sn, and O signals from the host lattice, indicating that doping does not cause appreciable phase separation or large dopant clusters.^60^ This distribution with La and Ce atoms either interstitially incorporated or substituted into the lattice is crucial for the controlled tuning of the material properties. The SEM micrographs show that all samples consist of nanoscale crystallites that form micrometer-scale aggregates, and the doped samples have more delicate and porous textures. Moreover, localized Sn enrichment is observed, consistent with the presence of a trace of SnO_2_ secondary phase. The combined compositional and morphological analyses; therefore, validate the retention of the Zn_2_SnO_4_ spinel structure, the successful incorporation of La and Ce dopants, and the existence of a minor secondary phase, providing a sound experimental basis for correlating structural features with functional performance.^61^

**Figure 8:**
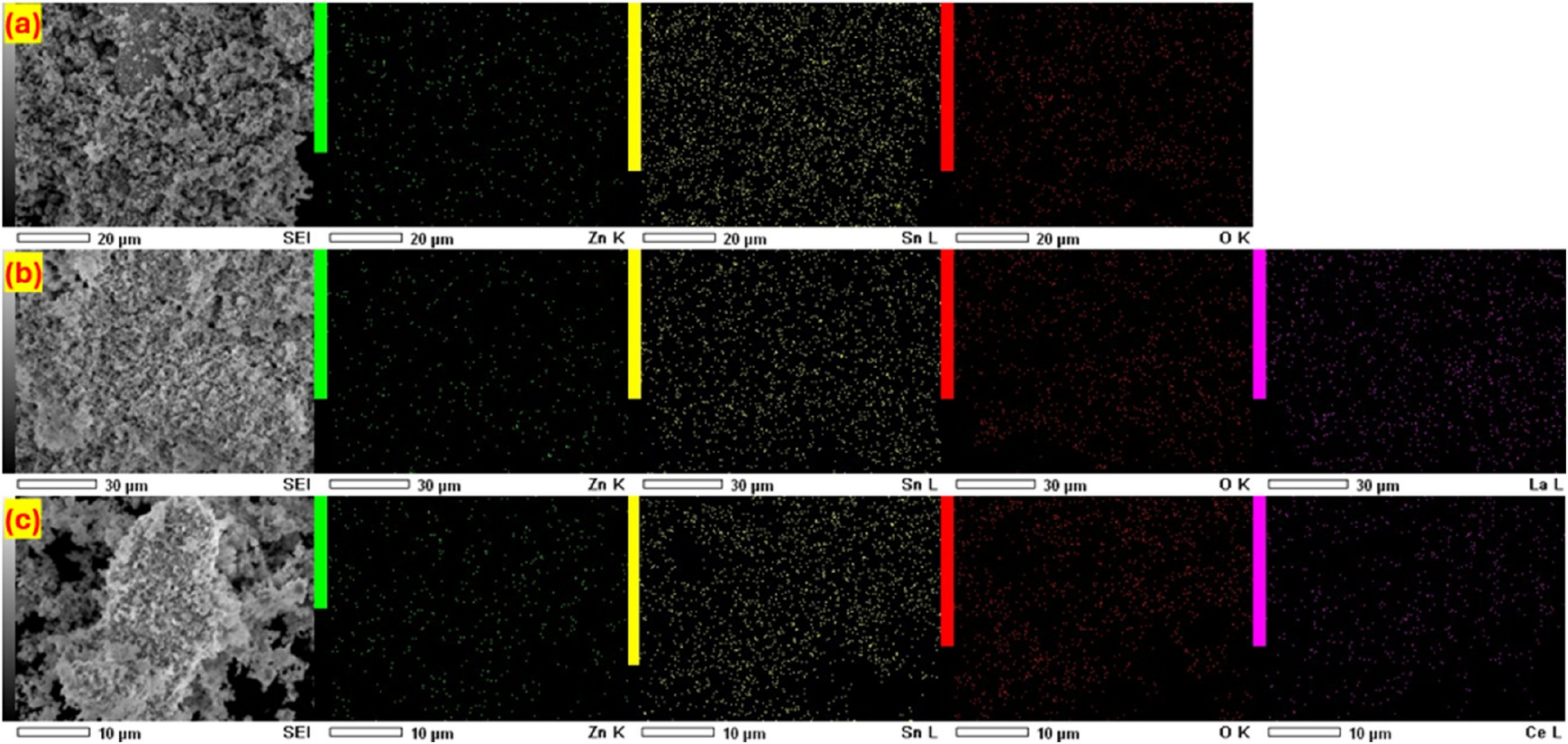
EDS mapping of (a) undoped, (b) La-doped, and (c) Ce-doped Zn_2_SnO_4_ spinel-type nanopowders.

### 3.6 XPS Analysis

XPS was performed to validate the elemental composition, oxidation states, and dopant-induced defect chemistry in doped samples, and the high-resolution spectra are presented in Figure 9a–f. The Zn 2p spectra (Figure 9a) show two sharp and symmetric peaks at 1021.3 (Zn 2p_3/2_) and 1044.5 eV (Zn 2p_1/2_), matching well with the standard Zn^2+^ binding energies, confirming that zinc exists predominantly in the +2 state in all samples. The narrow FWHM of these peaks indicates that La and Ce substitution does not introduce secondary Zn species or cause reduction of Zn, implying a stable Zn^2+^ framework after aliovalent doping. This supports the inference from XRD data that rare earth ions preferentially substitute the Sn sublattice rather than Zn sites in the spinel matrix.^62^ The Sn 3d spectra (Figure 9b) exhibit the typical spin–orbit pair at 486.8 (Sn 3d_5/2_) and 495.3 eV (Sn 3d_3/2_), consistent with Sn^4+^ in the Zn_2_SnO_4_ spinel environment. The absence of peaks around 484–485 eV confirms that no Sn^2+^ or metallic Sn^0^ species were formed during doping or high-temperature synthesis. Interestingly, the doped samples show a slight broadening of the Sn 3d peaks, suggesting local electronic perturbation, lattice strain, and defect formation induced by the charge imbalance created when La^3+^ or Ce^3+^/Ce^4+^ substitutes for Sn^4+^. This observation aligns with the microstrain and reduced crystallite size inferred from XRD, demonstrating that the rare earth dopants distort the SnO_6_ octahedral environment.^63^

**Figure 9:**
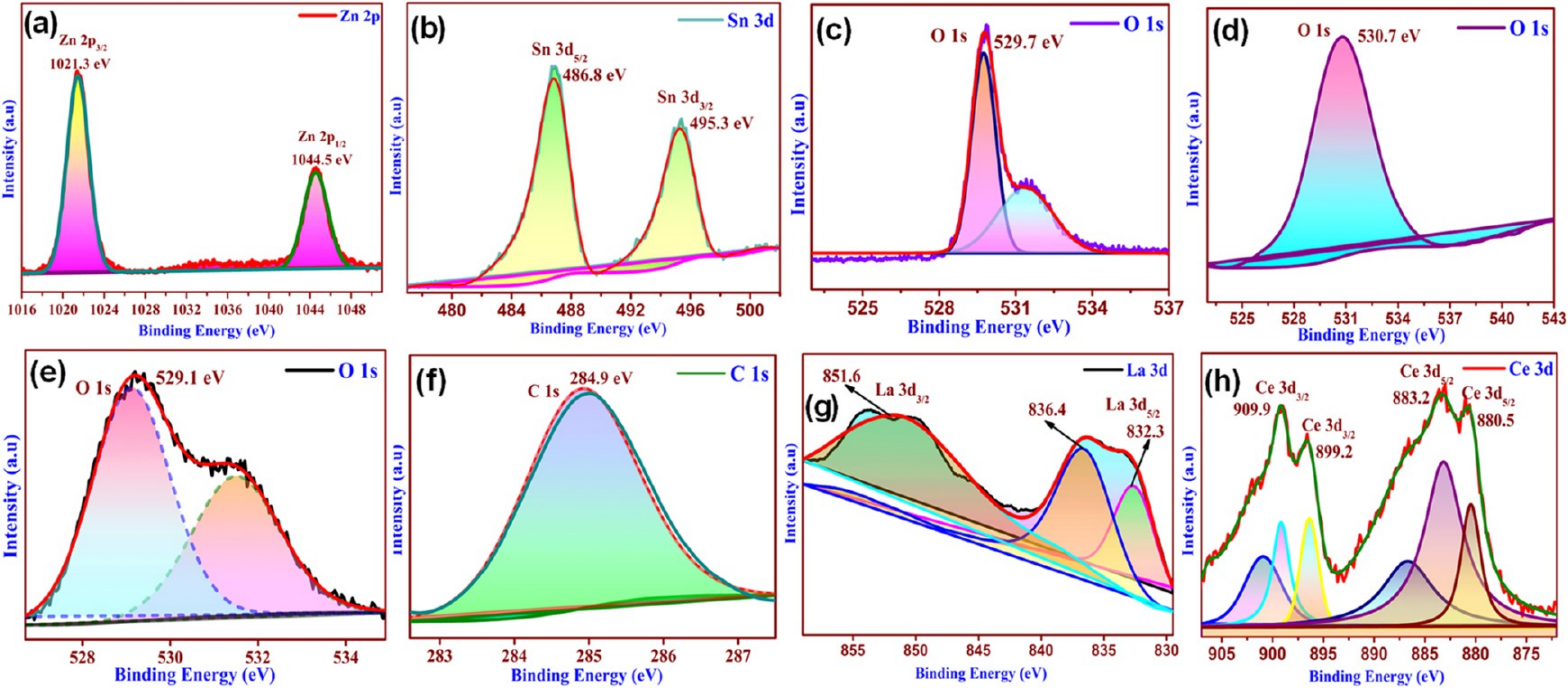
XPS spectra of La- and Ce-doped Zn_2_SnO_4_ spinel nanopowders: (a) Zn 2p, (b) Sn 3d, (c) O 1s (Undoped), (d) O 1s (La-doped), (e) O 1s (Ce-doped), (f) C 1s, (g) La 3d, and (h) Ce 3d.

The high-resolution O 1s XPS spectra of pure, La-doped, and Ce-doped Zn_2_SnO_4_ (Figure 9c–e) reveal a clear evolution of oxygen-vacancy-related defects with rare-earth substitution. In pristine Zn_2_SnO_4_ (Figure 9c), the O 1s peak is dominated by a lattice oxygen component (O_L_) at ∼529.6 to 529.8 eV, accompanied by a weaker high-binding-energy shoulder (O_V_) at ∼531.1 to 531.5 eV associated with oxygen-deficient environments. Upon La doping (Figure 9d), the O 1s spectrum becomes broader and more asymmetric, with a pronounced increase in the O_V_ contribution centered at ∼530.6 to 530.8 eV, indicating enhanced oxygen-vacancy formation due to aliovalent substitution of La^3+^ for Sn^4+^. The Ce-doped sample (Figure 9e) exhibits the strongest defect-related O_V_ component at ∼529.0 to 529.3 eV, reflecting the highest oxygen-vacancy concentration, likely facilitated by the mixed Ce^3+^/Ce^4+^ valence. The systematic increase of the O_V_ fraction from pure to La- and Ce-doped Zn_2_SnO_4_ is consistent with the Rietveld refinement and Williamson–Hall analyses, confirming dopant-induced lattice distortion and oxygen-vacancy-mediated defect formation, which underpin the observed changes in the optical and electronic properties. The C 1s peak (284.9 eV, Figure 9f) corresponding to adventitious carbon was used as an internal reference. Its consistent position and shape confirm that the charge correction across the samples is accurate. For the La-doped sample, the La 3d region (Figure 9g) shows well-defined peaks at 836.4 (La 3d_5/2_) and 851.6 eV (La 3d_3/2_), accompanied by characteristic shakeup satellite features, confirming the presence of La^3+^ in the lattice. Intensification of the defect-related O 1s component further supports the formation of oxygen vacancies associated with La incorporation, which is consistent with the observed orange PL emission, described later, because O vacancy-related states are known to promote deep-level transitions.^64,65^

The Ce 3d spectrum (Figure 9h) is more complex with multiple multiplet-split features at 899.2, 883.2, 909.9, and 880.5 eV, which collectively confirm the coexistence of Ce^3+^ and Ce^4+^ states with redox flexibility. Ce^3+^ enhances the concentration of oxygen vacancies, while Ce^4+^ helps maintain structural stability. This mixed valence also explains the strong green PL emission, described later, in the Ce-doped sample, because Ce^3+^-related electronic states and oxygen-vacancy complexes introduce multiple defect levels within the band gap.^66^ Thus, our XPS results provide a direct link from the dopant’s oxidation state to increased density of defect levels and eventually the PL color tunability. Taken together, the XPS results confirm successful incorporation of La^3+^ and Ce^3+^/Ce^4+^ into the Zn_2_SnO_4_ spinel lattice and provide strong evidence for dopant-induced lattice perturbation, oxygen vacancy formation, and defect-driven electronic modifications.^67^ These findings correlate closely with the XRD, vibrational (FTIR/Raman), optical (UV–vis), and PL results, validating the proposed defect engineering strategy and offering a comprehensive understanding of how rare earth substitution tailors the functional properties of Zn_2_SnO_4_ nanostructures.

To quantitatively evaluate the evolution of defect states, the O 1s XPS spectra were deconvoluted into lattice oxygen (O_L_) and defect-related oxygen (O_V_) components. As summarized in Table 4, the undoped Zn_2_SnO_4_ sample is dominated by the O_L_ contribution, with a comparatively lower fraction of O_V_. Upon La and Ce substitution, the relative area of the O_V_ component increases systematically, indicating an enhanced concentration of oxygen vacancies generated by aliovalent rare-earth substitution at Sn^4+^ sites. This quantitative increase in oxygen-vacancy-related states correlates directly with the observed band gap narrowing from UV–Vis analysis and with the enhancement of visible photoluminescence intensity. The higher O_V_ fraction in La- and Ce-doped samples confirms that charge-compensating oxygen vacancies introduce localized defect levels within the band gap, which act as efficient radiative recombination centers. In particular, the greater O_V_ contribution in Ce-doped Zn_2_SnO_4_ is consistent with the mixed Ce^3+^/Ce^4+^ redox chemistry observed in the Ce 3d spectra and explains the stronger green defect-related emission. This quantitative O 1s analysis establishes a clear relationship among oxygen-vacancy concentration (XPS), band structure modification (UV–Vis), and defect-mediated recombination pathways (PL), thereby providing strong experimental support for the proposed defect-engineering mechanism. The relative areas were obtained from Gaussian–Lorentzian fitting of the O 1s spectra, where the higher binding energy component (O_V_) systematically increases from undoped to La- and Ce-doped Zn_2_SnO_4_, confirming enhanced oxygen-vacancy formation upon aliovalent rare-earth substitution.

**Table 4:** Quantitative Deconvolution of O 1s XPS Spectra for Undoped, La-Doped, and Ce-Doped Zn_2_SnO_4_

Sample	O 1s component	Binding energy (eV)	Assignment	Relative area (%)
Zn_2_SnO_4_	O_L_	529.6–529.8	lattice oxygen (Zn–O/Sn–O)	73 ± 2
	O_V_	531.1–531.5	oxygen vacancies/defect oxygen	27 ± 2
Zn_2_SnO_4_:0.3La	O_L_	530.5–530.8	lattice oxygen	61 ± 2
	O_V_	531.8–532.2	oxygen vacancies/defect oxygen	39 ± 2
Zn_2_SnO_4_:0.3Ce	O_L_	529.0–529.3	lattice oxygen	57 ± 2
	O_V_	531.6–532.1	oxygen vacancies/defect oxygen	43 ± 2

### 3.7 PL Spectroscopy

PL spectra were analyzed to study the electronic structure, defect states, and radiative recombination paths in undoped and doped samples. The spectra were recorded at different excitation wavelengths (Zn_2_SnO_4_, 265 nm, Zn_2_La_0.3_Sn_0.7_O_4_, 392 nm, Zn_2_Ce_0.3_Sn_0.7_O_4_, 237 nm) to selectively probe the band-edge and defect-mediated transitions (Figure 10), which were intentionally selected to match the distinct optical absorption characteristics and defect-related electronic states of each composition. This excitation strategy enables efficient probing of the dominant band-edge and defect-mediated transitions specific to each sample rather than direct comparison of absolute emission intensities (Figure 10).^68^

**Figure 10:**
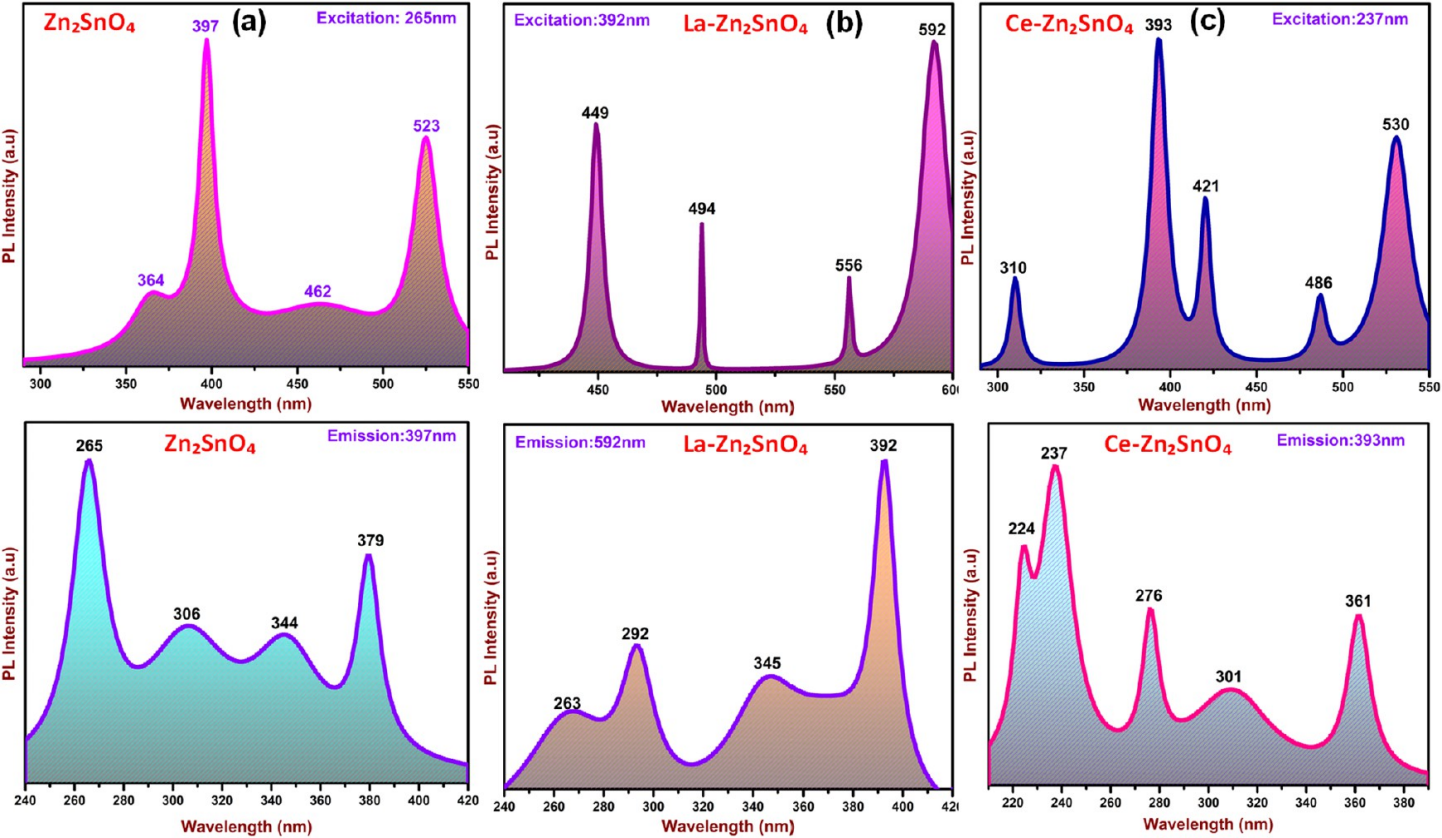
PL emission and excitation spectra of (a) undoped, (b) La-doped, and (c) Ce-doped Zn_2_SnO_4_ spinel-type nanopowders.

The PL response of undoped Zn_2_SnO_4_ is dominated by a near-UV/blue band at 397 nm, plus shoulders and defect-related bands at 364, 462, and 523 nm. The 397 nm feature arises from near-band edge excitonic recombination (electron–hole pair recombination or shallow donor–acceptor-type transitions) that is consistent with the wide band gap of spinel Zn_2_SnO_4_. The broader blue/green bands at 462 and 523 nm are attributed to radiative recombination via deep-level defect states, primarily oxygen vacancies (V_O_) and Sn- or Zn-associated localized states within the band gap.^69^ These defect bands are consistent with the XRD and SEM/EDS results showing nanoscale crystallites and some Sn-enriched surface regions (and trace quantities of SnO_2_), which increase the density of surface and near-surface trap states and lead to blue-green luminescence.

The emission profile of the La-doped sample is quite different from the other two. Under 392 nm excitation, there are discrete peaks at 449, 494, and 556 nm plus a strongly red-shifted peak at 592 nm. The prevailing emission at long wavelength (592 nm) signifies deep trap-mediated radiative recombination, indicating the formation of deeper energy levels in the band gap upon La introduction. La^3+^ itself does not exhibit luminescence under self-excitation with visible light, because it has no intense f–f transitions in the visible region. Therefore, this intense 592 nm luminescence must arise from host-related defect centers, whose levels are stabilized or created by dopant incorporation and by the charge-compensating processes (presumably oxygen vacancies) required when La^3+^ replaces Sn^4+^. The increase in intensity of long-wavelength defect luminescence and FWHM is diagnostic of increased nonstoichiometry, elevated microstrain, and higher radiative trap concentration, as expected from the lattice expansion (broadened XRD peaks) and more open structure with a larger surface area (as seen in SEM).

The Ce-doped sample has a complex PL spectrum with multiple peaks: strong features at 310 and 393 nm and a number of blue-green bands at 421, 486, and 530 nm. Ce is redox active because it exists as Ce^3+^ and Ce^4+^, and Ce^3+^ has allowed 5d → 4f transitions that cause structured near-UV/blue emission. Thus, the near-UV/blue bands (especially 310–393 nm) are presumably a mixture of host excitonic emission and Ce^3+^-associated 5d → 4f luminescence, while the blue-green bands (421–530 nm) are primarily defect-mediated. Ce also promotes the formation of mixed-valence oxygen vacancy, which raises the defect center concentration and affects local lattice relaxation,^70^ thereby justifying the complex spectrum, relatively broad bands, and relatively small Scherrer crystallite size indicated by XRD. Overall, the Ce-doped sample displays both dopant-related emission (potential signatures of Ce^3+^) and strong defect-related luminescence.

The intensity ratios, peak shifts, and bandwidths of the three samples provide useful mechanistic insights. After La or Ce doping, the inhibition/redistribution of near-band-edge emission to longer-wavelength defect emission indicates that doping increases the density of midgap radiative states. These states are radiative traps (as suggested by the strong PL bands), and they can either prolong the carrier lifetime (beneficial for surface reactions) or promote nonradiative recombination losses if nonradiative processes are dominant.^71^ These PL data thus support the structural features from XRD/SEM analyses: aliovalent rare-earth substitution causes lattice expansion, increases microstrain, and elevates oxygen vacancy concentration, all of which influence the PL behavior and functional properties.

The combined XPS, UV–Vis, and PL analyses confirm that the optical response of RE-modified Zn_2_SnO_4_ is governed by defect-mediated electronic states intrinsic to the spinel lattice. XPS O 1s spectra show enhanced oxygen-vacancy-related components in La- and Ce-doped samples, indicating that aliovalent substitution at Sn^4+^ sites generates charge-compensating defects and localized electronic levels. These defect states correlate directly with the observed band gap narrowing and the evolution of PL from near-band-edge emission in undoped Zn_2_SnO_4_ to strong, dopant-selective visible luminescence in La- and Ce-doped samples. The orange emission in La-doped Zn_2_SnO_4_ arises from deep-level defect states stabilized by La^3+^ substitution, whereas the green emission in Ce-doped Zn_2_SnO_4_ originates from oxygen-vacancy-related states and Ce^3+^-associated electronic transitions. Although a minor SnO_2_ phase is detected by XRD, similar Zn_2_SnO_4_/SnO_2_ coexistence has been widely reported in defect-engineered systems and does not dominate the optical response.^29–31^ Bulk SnO_2_ typically exhibits weak or near-UV emission and cannot account for the strong, dopant-dependent visible PL observed here. Compared with other AB_2_O_4_ spinels such as NiFe_2_O_4_ thin films and NiFe_2_O_4_–ZrC composites, where defect-driven luminescence is accompanied by narrower band gaps and magnetic losses.^18,19^ Zn_2_SnO_4_ uniquely combines a wide band gap, high electron mobility, and optical transparency with controlled defect-assisted visible emission. These results establish rare-earth-modified Zn_2_SnO_4_ as a superior defect-engineered AB_2_O_4_ spinel for optoelectronic and photonic applications.

### 3.8 CIE Chromaticity and Color Tuning

The PL spectra were integrated to determine the CIE 1931 chromaticity coordinates of the undoped and doped samples. According to Figure 11, the measured coordinates and perceived colors are undoped Zn_2_SnO_4_ (*x* = 0.1363, *y* = 0.3524, violet), Zn_2_SnO_4_:0.3La (*x* = 0.4042, *y* = 0.3079, yellowish orange), and Zn_2_SnO_4_:0.3Ce (*x* = 0.1534, *y* = 0.5047, green). These coordinates clarify how the relative intensities and spectral positions of several PL bands are combined to create a single perceived color. The undoped sample has its CIE position in violet, indicating dominant near-UV/blue band-edge emission and minor shoulders of blue-green defect emission.^72^ La doping dramatically shifts the chromaticity into the yellow-orange quadrant owing to the dominant ∼592 nm defect band, demonstrating how deep traps can convert a UV/blue emitter into a visible orange phosphor. In contrast, Ce doping enhances relative emission through the green spectral window (420–540 nm) owing to defect bands and possible Ce^3+^ transitions, changing the chromaticity into the green region. These color changes illustrate that spinel Zn_2_SnO_4_ can be converted into a color-tunable phosphor by aliovalent doping and defect engineering under controlled conditions without using conventional activator ions. Such tunability is potentially useful for phosphors, lighting, and display technologies where the emission color must be managed.^73,74^ In Table 5 summarizes the representative PL peak positions, estimated FWHM, peak assignments, while Table 6 presents the key findings and defect correlation for undoped, La-doped, and Ce-doped Zn_2_SnO_4_ samples.

**Figure 11:**
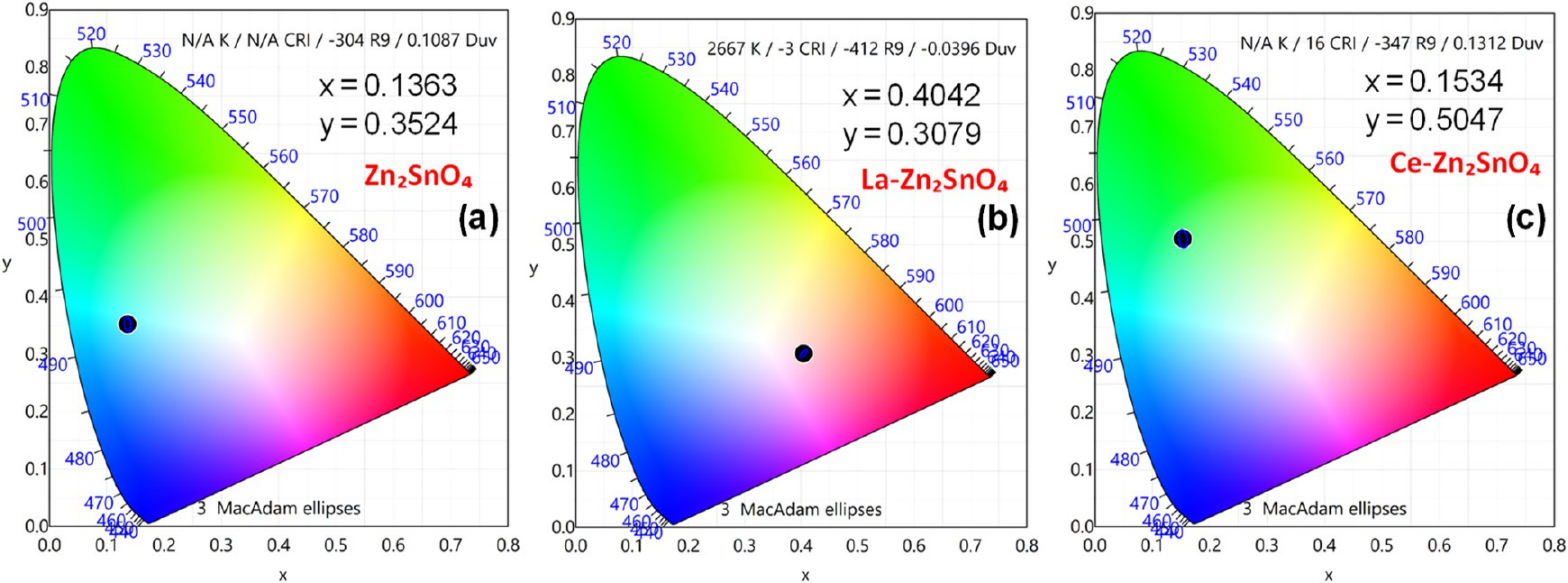
CIE diagrams of (a) undoped, (b) La-doped, and (c) Ce-doped Zn_2_SnO_4_ spinel-type nanopowders.

**Table 5:** Representative PL Peak Positions, Estimated FWHM, and Peak Assignments for Undoped, La-Doped, and Ce-Doped Zn_2_SnO_4_ Samples

Sample	Peak center (nm)	Estimated FWHM (nm)	Tentative assignment
Zn_2_SnO_4_	364	18	near-band-edge/shallow exciton or shallow donor–acceptor transition
	397	20	near-band-edge excitonic recombination
	462	28	blue defect emission due to oxygen vacancies/Sn-related deep levels
	523	32	green defect emission due to deep donor–acceptor pair transitions (V_O_, cation defects)
Zn_2_SnO_4_:0.3La	449	22	defect-related emission due to shallow/medium trap states stabilized by La
	494	24	defect/trap emission (donor–acceptor pairs)
	556	28	deep defect center (oxygen-vacancy complexes or dopant-vacancy complexes)
	592	34	strong deep-level emission due to dopant-stabilized deep traps (responsible for yellow/orange color)
Zn_2_SnO_4_:0.3Ce	310	15	near-UV emission due to possible Ce^3+^ 5d → 4f contribution and/or host excitonic emission
	393	20	band-edge/Ce^3+^-related emission (mixed contribution)
	421	26	blue defect emission due to oxygen vacancies/Ce-vacancy complexes
	486	30	blue-green defect band due to deep traps (V_O_, cation defects)
	530	32	green defect emission contributes to green chromaticity

**Table 6:** Key Findings and Defect Correlation for Undoped, La-Doped, and Ce-Doped Zn_2_SnO_4_ Samples

Sample	Dominant emission	Primary assignment	CIE color
Undoped	near-UV/blue (397 nm)	near-band-edge excitonic recombination	Violet (*x* = 0.14, *y* = 0.35)
La-doped	strong deep-level (592 nm)	dopant-stabilized deep traps (oxygen vacancies)	Yellowish orange (*x* = 0.40, *y* = 0.31)
Ce-doped	complex multipeaked (310–530 nm)	mixed Ce^3+^ (5d–4f) and defect-mediated (oxygen vacancies, Zn/Sn interstitials and complexes)	Green (*x* = 0.15, *y* = 0.50)

In summary, La and Ce substitution in Zn_2_SnO_4_ causes dopant-specific, strong changes in the emission spectra. La stabilizes deep trap states to create a strong yellow-orange emission, while Ce introduces a rich mixture of Ce-related and defect-mediated emissions that lead to green chromaticity. These alterations are fully consistent with the lattice expansion determined by XRD, peak broadening, and dopant incorporation and defect formation, as confirmed by SEM/EDS.

## 4 Conclusion

La- and Ce-doped Zn_2_SnO_4_ spinels were successfully synthesized via a solid-state route, demonstrating that aliovalent rare-earth substitution can effectively tailor the structural and optoelectronic properties while maintaining the cubic spinel structure. Dopant-induced lattice distortion and oxygen-vacancy formation, progressive band gap narrowing from 3.57 eV (undoped) to 3.16 eV (La-doped) and 3.12 eV (Ce-doped), and clear dopant-selective photoluminescence with orange and green emissions for La- and Ce-doped Zn_2_SnO_4_, respectively. These results establish a direct structure–defect–optical correlation and highlight rare-earth-modified Zn_2_SnO_4_ as a promising multifunctional material for visible-light optoelectronic, luminescent, and optical sensing applications.

## Data Availability

The data underlying this study are not publicly available due to ethical and privacy considerations related to participant confidentiality. The data are available from the corresponding author upon reasonable request, subject to approval by the relevant institutional review board and compliance with applicable data protection regulations.
